# Brodmann’s Area Template Based Region of Interest Setting and Probabilistic Pathway Map Generation in Diffusion Tensor Tractography: Application to the Arcuate Fasciculus Fiber Tract in the Human Brain

**DOI:** 10.3389/fnana.2016.00004

**Published:** 2016-01-21

**Authors:** Dong-Hoon Lee, Do-Wan Lee, Bong-Soo Han

**Affiliations:** ^1^Division of MR Research, Department of Radiology, Johns Hopkins University School of MedicineBaltimore, MD, USA; ^2^Department of Radiological Science, College of Health Science, Yonsei UniversityWonju, South Korea

**Keywords:** Brodmann’s area, region of interest, diffusion tensor tractography, arcuate fasciculus, probabilistic pathway map

## Abstract

The purpose of this study is to acquire accurate diffusion tensor tractography (DTT) results for arcuate fasciculus (AF) fiber tract using Brodmann’s area (BA) template for region of interest (ROI) setting. Thirteen healthy subjects were participated in this study. Fractional anisotropy (FA) map of each subject was calculated using diffusion tensor data, and T1w template was co-registered to FA map. The BA template was also co-registered using the transformation matrix. The ROIs were drawn in the co-registered BA template, and AF fiber tract was extracted. To generate the probabilistic pathway map, a binary mask image was generated based on the fiber tract image and co-registered to T1w template image. We also measured relative location of the AF fiber tract. The location of the probabilistic pathway map of each subject’s AF fiber tract was well defined in the brain. By using this probabilistic map, the mediolateral position ratio of AF was measured 18%, and the anteroposterior position ratio of AF was measured 35%, respectively. This study demonstrated that the AF fiber tract can be extracted using BA template for ROI setting and probabilistic pathway of fiber tract. Our results and analytical approaches can helpful for accurate fiber tracking and application of perspective clinical researches.

## Introduction

Magnetic resonance diffusion tensor tractography (DTT) is widely used for neural fiber tracking and analysis of specific fiber tract. The important portion of the DTT application to *in vivo* is how to set the region of interest (ROI) for the DTT analysis process. Many researchers have set ROIs based on the anatomical image and the calculated color-coded fractional anisotropy (FA) map according to their research purpose, generally (Hong et al., 2009; Kim and Jang, 2013; Li et al., 2013). Although multi-ROIs based analysis is used for these approaches, they have a drawback in terms of accuracy of the results because of an user-dependent ROI settings. To overcome this drawback, the functional MRI (fMRI) activation results have been combined with DTT analysis (Propper et al., 2010; Li et al., 2013). This combination approach provides accurate ROI setting than a manual ROI setting. However, the sizes of fMRI activation areas could be possibly changed according to the given statistically significant value and the additional image acquisition process causing time consumption.

In this study, we applied the Brodmann’s area (BA) template to set ROIs for accurate DTT analysis for arcuate fasciculus (AF) fiber tract. Among the multiple neural fiber tracts in the human brain, the AF is an important neural fiber tract which is connecting the frontal (Broca’s) and temporal (Wernicke’s) areas, and it has been associated with language functions. Thus, lesions to the AF caused various types of language problems such as conduction aphasia and speech deficits (Yamada et al., 2007; Jang, 2013; Li et al., 2013). Therefore, identification of the anatomical characteristics with its location of AF fiber tract in the normal human brain or in patients with aphasia is became an important issue because it would be helpful to neuroscientists or clinical researchers to predict the neural fiber recovery status for aphasia, and follow-up studies. Moreover, the BA template is a kind of standard template, which demonstrates regions of the human cortex divided into 46 areas based on cytoarchitectural features (Thottakara et al., 2006). Applying the characteristics of BA template for divided cortex regions as a standard, our analytical approaches provides accurate and helpful ROI setting for DTT studies. In addition, we generated fiber tract probability map of AF to estimate the fiber tract pathway in the brain.

## Materials and Methods

### Subjects

Thirteen healthy subjects, nine men and four women, were participated in this study (right handed, mean age: 38.7 ± 6.4 years, age range: 26–50 years). They had no previous history of neurological or physical disease. All participants underwent evaluation by radiologist and neurologist, and they were diagnosed as normal subjects. All subjects understood the study purpose, and provided written informed consent. This study protocol was approved by the local Institutional Review Board.

### Data Acquisition and Analysis

Diffusion tensor imaging (DTI) data were acquired using a 1.5 T MR scanner (Gyroscan Intera, Philips Healthcare, The Best, Netherlands) with a six-channel phased array sensitivity encoding (SENSE) head coil using a single-shot spin echo echo-planar imaging (EPI) pulse sequence. The DTI data were acquired with the following parameters: time of repetition (TR)/time of echo (TE) = 10,726/75 ms, field of view (FOV) = 221 mm, acquisition matrix = 96 × 96, reconstruction matrix = 128 × 128, slice thickness = 2.3 mm, and SENSE factor = 2. Diffusion weighting was applied along 32 non-collinear and non-coplanar diffusion sensitizing gradients with a *b*-value of 1000 s/mm^2^. We acquired 67 contiguous transverse slices covering the entire brain with no slice gaps, and interleaved slice acquisition was applied to minimize the cross-talk caused by no gap between the slices.

**Figure 1:**
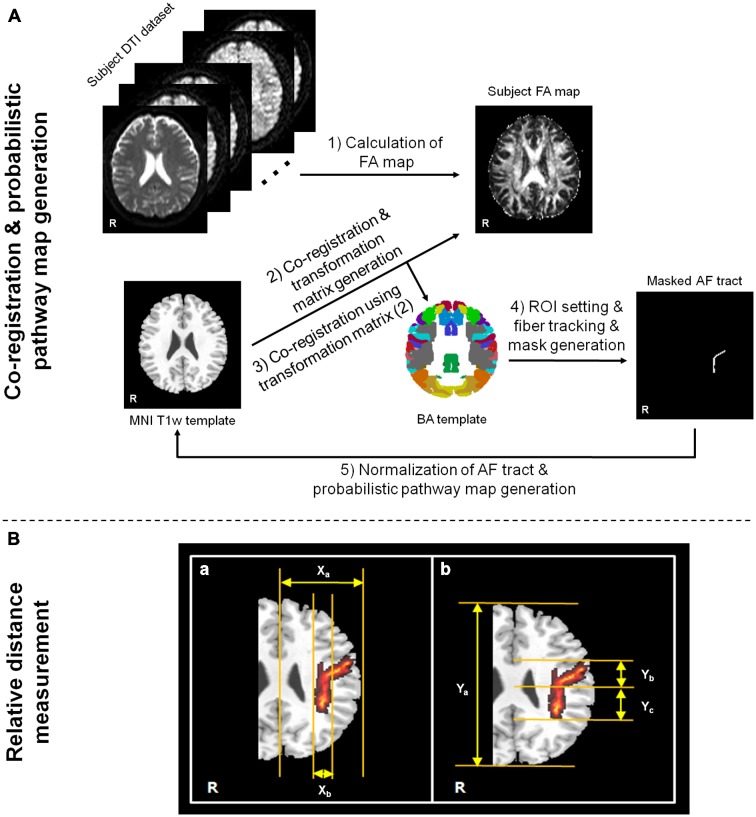
**Flow chart of data analysis procedures for ROI setting in fiber tracking/probabilistic pathway generation (A) and measurement process of relative fiber locations (B).** For the arcuate fasciculus (AF) relative location measurement in probabilistic pathway map, **(a)** indicates the method of mediolateral location ratio measurement between X_a_ and X_b_, and **(b)** indicates the method of anteroposterior portion ratio measurement between Y_a_ and Y_b_ or Y_c_. The measurement processes of location ratios were performed in the Montreal Neurological Institute (MNI) T1w template, which is presented on the corona radiata location.

Before the DTI data analysis, the effects of eddy currents and head motion were corrected by registering all DWI images to non-diffusion weighted images (*b*-value = 0 s/mm^2^) using affine multi-scale registration by FSL (Smith et al., 2004)^1^. DTI Studio software (Department of Radiology, Johns Hopkins University School of Medicine, Baltimore, MD, USA), which was fiber assignment by the continuous tracking (FACT) algorithm and a multiple ROIs approach, was used for the calculation of diffusion parameter maps and fiber tracking (Wakana et al., 2004; Jiang et al., 2006). To extract and evaluate the AF fiber tract from each subject, we used two standard brain templates [BA template and Montreal Neurological Institute (MNI) T1w template], which were provided in MRIcro software^2^. The BA template provides a volume mask that is subdivided into 46 discrete cortical regions, each representing a different BA area. While we use the pre-defined cortical regions in the BA template, we can simply select or draw the specific region area, which is correlated with the origin of neural fiber tract, for ROI selection in the fiber tracking. In this way, it is possible to minimize the erroneous factor induced by user-dependent ROI setting. In addition, the MNI T1w template was used for the brain image normalization process. All DTI datasets acquired from MRI scanner for each subject and calculated FA map have slightly different orientation and location information. Therefore, some erroneous factors that were induced from different structures and/or locations between subjects can be prevented by using the brain normalization process. Moreover, the normalization process based on the template image for all datasets is more helpful to generate the probability pathway map of fiber tract in order to maintain the consistency of the locations. In this study, the AF fiber tract was analyzed only in the dominant (left) hemisphere from all subjects. The flow chart of processing procedures was shown in Figure 1A, which was performed in the following orders: (i) Subject FA map was calculated using DTI Studio software; (ii) The T1w template was co-registered to the each subject’s FA map using SPM8 (Wellcome Department of Cognitive Neurology, London, UK) software. Due to the small difference in the image contrast between the FA map and the T1w template, it is possible to minimize the erroneous factor in the co-registration process; (iii) To perform the normalization process between the BA template and the diffusion tensor datasets, the transformation matrix, which was generated on step (ii), was applied to BA template; (iv) Two ROIs were drawn in the Broca’s area and Wernicke’s area based on the normalized BA template, and the AF fiber tract of each subject was extracted with a following criteria; a voxel with the FA value lower than 0.2 or the trajectory angle lower than 80 degrees; and (v) Binary masks of the extracted fiber tract for each subject were generated. The binary masks only have two values; one (voxels for fiber tract location) and zero (voxels for non fiber tract location). The masks of all the subjects were normalized using the MNI T1w template from MRIcro with a 12 parameter affine registration using inverse transformation matrix of the original co-registration process. These normalized fiber tract masks were summed, and divided by the total number of subjects to generate the probabilistic pathway map of AF. The probabilistic pathway map was overlaid on the MNI T1w template with a different scale according to the probability value of a voxel.

To investigate the location of the AF fiber tract pathway on each subject, the relative location was measured by the occupied ratio of AF tract at the basis of left hemisphere. The measurement processes were performed with mediolateral part and anteroposterior part using the generated probabilistic pathway of AF fiber tract on the MNI T1w template (Figure 1B). The mediolateral location was measured by the ratio between the length from the longitudinal fissure to most lateral boundary of left cerebral hemisphere (X_a_) and the length from the medial to lateral location of horizontal part of AF (X_b_) as follows: (X_a_/X_b_) × 100. The anteroposterior location ratio was measured between the length from the most anterior boundary to the most posterior boundary (Y_a_) and the length from the anterior to posterior boundary of vertical part of AF (Y_b_ or Y_c_). The anteroposterior location ratio, more detailed, was measured separately based on the confluence of horizontal part (ratio between Y_a_ and Y_b_) and vertical part (ratio between Y_a_ and Y_c_) as follows: (Y_a_/Y_b_) × 100 and (Y_a_/Y_c_) × 100.

## Results

**Figure 2:**
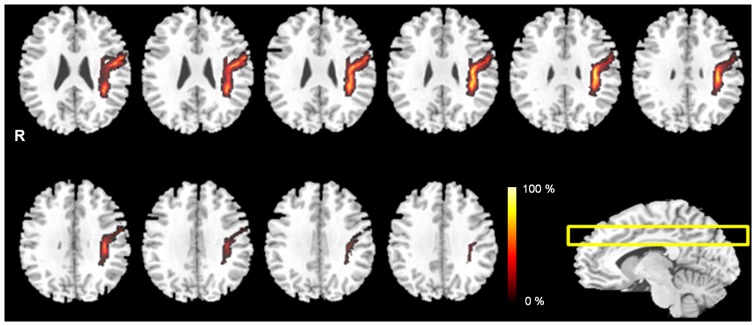
**The probabilistic pathway maps of AF fiber tract in the multi-slice locations.** The color-scale bar indicates the probabilistic values of the fiber tract.

The probabilistic pathway maps of AF fiber tract for all subjects group are shown in Figure 2. The color-scale range indicates the probability of a voxel being part of the AF fiber tract pathway. In this probabilistic pathway map of AF fiber tract, the measured mediolateral position ratio was 18%. The measured ratio of the anteroposterior position was 35% based on the AF curvature point. The ratio was measured with the upper portion 15% and the lower portion 20%, respectively. Based on the results, the measured mediolateral portion of AF is accounted for 1/5 of the total mediolateral length of the hemisphere on the MNI T1w template. The measured anteroposterior portion of AF was 1/2 length compared with total anteroposterior length of the hemisphere. In addition, the extracted AF fiber tract that was overlaid on the transverse image plane of the MNI T1w template has not been fully shown the overall shape of AF fiber tract structure because of the characteristic of curved shape of *in vivo* AF fiber tract; however, notably, extracted AF fiber tract for each subject, which was created by mask image in the analysis procedure (Figure 1), and reconstructed probability map (Figure 2) described that the AF fiber tract from our results was connected two brain regions between the Broca’s area in the inferior frontal gyrus and Wernicke’s area in the posterior superior temporal gyrus. Furthermore, the generated probabilistic pathway map clearly showed that the distributions of combined AF fiber tract, which was extracted by BA template for ROI setting from each subject, was well located and defined in the human brain without any dislocation errors.

## Discussion

The BA is well-defined human brain cortex regions of 46 areas according to their unique functions. Many researchers have used the BA template to indicate specific locations of brain activity in their studies such as patients with neurological disease or treatment strategies because the BA differentiate regions not only anatomically distinct but functionally as well. Especially, among the BA, Broca’s area and Wernicke’s area that are connected via the AF neural fiber tract that is curving around the sylvian fissure that links the temporal and frontal language areas (Rilling et al., 2008; Jang, 2013; Tak and Jang, 2014). The AF could be expected to be the most important fiber tract associated with language functions, and it is has a distinct shape of a curve combined different directions unlike other fiber tracts such as corticospinal tract (representative superior-inferior fiber direction) or corpus callosum (representative left-right fiber direction). Since the AF fiber tract is highly associated with the patients who have hampered language skills such as aphasia, identification of the accurate location of AF fiber tract is significantly considered in the clinical perspective. Many of the approaches with multi-modality imaging techniques and/or invasive intra-operative surgery has been performed to find the neuroanatomical characteristics of AF fiber tract and to evaluate the critical role for the feed-forward and feedback control of language production (Duffau et al., 2002; Breier et al., 2008; Hosomi et al., 2009; Marchina et al., 2011; Zhao et al., 2012; Yamao et al., 2014). The DTT method, which has been introduced in the past to track the neural fiber tract, is widely used to demonstrate the neural fiber characteristics using calculated diffusion phenomena of the *in vivo* water molecules. This approach is adequate for the fiber tract visualization, as well as the easy to application. Due to these characteristics, the DTT method and its technical development allowed visualization of white matter associated fiber tracts *in vivo*. However, although the evaluation of fiber tracts was progressed with the DTT method, there is a limitation still remained due to the accuracy of ROI setting in DTT.

In this study, we employed the non user-dependent ROI setting for DTT based on the BA template. The ROIs defined from BA template have an advantage for consistency of neural fiber tract in comparison with user-dependant ROI settings. In addition, we normalized individual AF tract to MNI T1w template to investigate the tendency of AF location and its probabilistic pathway in the human brain. The probabilistic pathway could provide better estimation of the fiber tract connection probabilities for a group of subjects. Up to now, the ROI selection procedures for DTT analysis were generally performed with user-dependent ROI setting, and it may cause analytical errors in parts of the identity and reproducibility even though the ROI was well defined by experienced researchers. Notably, the overall proposed analysis approach for *in vivo* human neural fiber tracking, which performed with the ROI selection based on the BA template, has analytical strength to allow the acquisition for more accurate fiber tracts regardless of any contamination of ROI setting errors by users or researchers. In terms of maintaining the identify and reproducibility of the results, it can be provided the high agreements because of the two main analytical procedures such as brain normalization and ROI area extraction from BA template without any manual settings. Furthermore, our approaches could easily be adapted to analysis for DTT studies, and lead to fiber connection analysis of BA in other brain areas accurately.

There are some limitations of this study. First, we have a limitation to our DTT analysis procedure due to the consideration of the deterministic fiber tracking algorithm. Therefore, we believe that the application of other fiber tracking algorithms based on the probabilistic fiber tracking algorithm with BA template and comparison studies will be provided with more helpful information to evaluate the BA template based ROI setting in clinical researches. Second, for the subject recruitment, we only considered normal subjects with relatively low populations. In the future study, with a large number of subjects and/or patients who had diseases in the AF fiber tract will be participated, we believe that the results also give more reliability.

In conclusion, we demonstrated the AF fiber tracking with the BA template for ROI selection and its probabilistic pathway in the human brain. We believe that our proposed analytical approaches are sufficiently extended to other DTT studies for ROI setting, and these may be provided accurate neural fiber tract information and clinical research settings.

## Author Contributions

D-HL, D-WL and B-SH designed and coordinated the study. D-HL and B-SH acquired the data. D-HL and D-WL analyzed the data. D-HL drafted the manuscript. B-SH mentored the study.

## Conflict of Interest Statement

The authors declare that the research was conducted in the absence of any commercial or financial relationships that could be construed as a potential conflict of interest.

## Footnotes

^1^www.fmrib.ox.ac.uk/fsl, Analysis Group, FMRIB, Oxford, UK.

^2^www.mricro.com, Chris Rorden, CA, USA.

## Abbreviations

BA: Brodmann’s area
ROI: region of interest
AF: arcuate fasciculus
DTT: diffusion tensor tractography
DTI: diffusion tensor imaging
MNI: Montreal Neurological Institute
FA: fractional anisotropy.
